# High-Altitude Wild Species *Solanum arcanum* LA385—A Potential Source for Improvement of Plant Growth and Photosynthetic Performance at Suboptimal Temperatures

**DOI:** 10.3389/fpls.2019.01163

**Published:** 2019-09-24

**Authors:** Quy-Dung Dinh, Annemarie Dechesne, Heleen Furrer, Graham Taylor, Richard G. F. Visser, Jeremy Harbinson, Luisa M. Trindade

**Affiliations:** ^1^Plant Breeding, Wageningen University and Research, Wageningen, Netherlands; ^2^Graduate School Experimental Plant Sciences, Wageningen University and Research, Wageningen, Netherlands; ^3^Horticulture and Product Physiology Group, Wageningen University and Research, Wageningen, Netherlands

**Keywords:** suboptimal temperature, photosynthesis, dry matter partitioning, sucrose metabolism, *Solanum lycopersicum*, *Solanum arcanum*

## Abstract

Plant growth, development, and yield of current tomato cultivars are directly affected by low temperatures. Although wild tomato species have been suggested as a potential source for low temperature tolerance, very little is known about their behavior during the reproductive phase. Here, we investigated the impact of suboptimal temperatures (SOT, 16/14°C), as compared to control temperatures (CT, 22/20°C), on plant growth, photosynthetic capacity, and carbohydrate metabolism. Under these conditions, two genotypes were analyzed: a *Solanum lycopersicum* cultivar Moneymaker and a high-altitude wild species *Solanum arcanum* LA385, from flowering onset until a later stage of fruit development. Total dry matter production in cv. Moneymaker was reduced up to 30% at SOT, whereas it was hardly affected in wild accession LA385. Specific leaf area, total leaf area, and number of fruits were also decreased at SOT in cv. Moneymaker. In contrast, wild accession LA385 showed an acclimation to SOT, in which Φ_PSII_ and net CO_2_ assimilation rates were less affected; a similar specific leaf area; higher total leaf area; and higher number of fruits compared to those at CT. In addition, LA385 appeared to have a more distinct sucrose metabolism than cv. Moneymaker at both temperatures, in which it had higher contents of sucrose-6-phosphate, sucrose, and ratio of sucrose: starch in leaves and higher ratio of sucrose: hexose in fruits. Overall, our findings indicate that wild accession LA385 is able to acclimate well to SOT during the reproductive phase, whereas growth and development of cv. Moneymaker is reduced at SOT.

## Introduction

The growth, development, and yield of crops, such as tomato, are directly influenced by temperature among other climatic factors such as light, water, and humidity. Tomato plants and their fruits can suffer physiological injury when exposed for several weeks to non-freezing temperature below 12°C (Brüggemann et al., 1992, Heuvelink and Dorais, 2005). It is reported that the mature tomato plants cannot recover after being exposed to 4°C for 10 days (Patade et al., 2018). It is therefore difficult to grow tomatoes commercially as a field crop in the Netherlands, and other countries with cool-temperate climate where temperatures below 12°C are common in most seasons of the year. In a cool-temperate climate, tomatoes are normally commercially grown inside heated glasshouses where growing conditions are tightly controlled for optimal growth, leading to high yields. For example, in the Netherlands, the average yield of tomatoes produced in heated glasshouses is about 70 kg m^−2^ yr^−1^ (Netherlands Foreign Investment Agency, 2017). This high yield is obtained at a temperature of 19–20°C which is considered to be the economic optimum temperature for heated-glasshouse tomato cultivation in the Netherlands (Van Der Ploeg and Heuvelink, 2005). However, this high productivity requires a large energy input, which accounts for one third of the total production cost and raises concerns over the environmental impact of this production system. It is estimated that a temperature decrease of 2°C inside the glasshouse would reduce energy use for heating by 16% (Elings et al., 2005). Therefore, it is desirable to decrease the temperature set point in the glasshouse so that energy costs and negative environment impacts can be reduced. With modern greenhouse tomato cultivars, such a temperature decrease would result in a decrease in plant growth and yield, something which would make the otherwise desirable reduction in energy use economically unsustainable. For greenhouse tomatoes in the Netherlands, temperatures below the economic optimum temperature 19–20°C, but above the threshold temperature for chilling injury (12°C), are classified as suboptimal temperatures (SOT).

Suboptimal temperatures are reported to have several negative effects on the growth and development of cultivated tomatoes. It has been shown that SOT decreased the initiation rate of new leaves, the leaf-area ratio, and the specific leaf area in young cultivated tomato plants, leading to a lower relative growth rate (Hoek et al., 1993, Venema et al., 1999b, Heuvelink, 2005). In addition, several tomato cultivars exposed to SOT increased leaf soluble sugars and starch content during their vegetative and reproductive phases (Venema et al., 1999b, Kläring et al., 2015). In the reproductive stage, low temperatures cause a delay in the time to flowering and the first fruit harvest (Hurd and Graves, 1985, Kläring et al., 2015), a reduction in fruit set due to poor pollen quality (Picken, 1984, Zinn et al., 2010), and also parthenocarpic fruits (Adams et al., 2001).

Variation in the SOT response between cultivated tomatoes is limited, and this hinders breeding for improved levels of production at lower temperatures. The limited genetic variation found in cultivated tomatoes for low temperature tolerance could be augmented by using germplasm from high-altitude wild tomatoes species (Venema et al., 1999a, Venema et al., 1999b, Foolad and Lin, 2000, Venema et al., 2005, Van Der Ploeg et al., 2007a, Van Der Ploeg et al., 2007b). While high-altitude tomato accessions have been shown to have better low temperature tolerance, all these studies have been carried out with young plants in the vegetative growth stage, even though the reproductive phase of tomatoes is known to be the most temperature-sensitive growth stage (Zinn et al., 2010). The objective of the study we report here was therefore to investigate the differential responses to SOT of photosynthesis, growth, and carbohydrate distribution between different plant organs from flowering onset until fruit harvest between a cultivated and a high-altitude wild species. The findings from this study provides new insights into how this wild tomato *Solanum arcanum* LA385 copes with SOT in those aspects during the reproductive stage. The knowledge from this study would bring us one step closer to exploit this wild tomato as the source to breed for new cultivar performing well at SOT, which would ultimately help the tomato greenhouse cultivation in temperate regions reduce energy consumption and be more sustainable.

## Materials and Methods

### Plant Materials

Two genotypes were used in this study: a cultivated tomato *Solanum lycopersicum* cv. Moneymaker (Moneymaker or MM) and a high-altitude *S. arcanum* LA385 (LA385). The seeds were obtained from Centre of Genetic Resources, Wageningen, the Netherlands. Cultivar Moneymaker is an indeterminate and self-compatible tomato type. It is a popular English greenhouse variety which was first released in 1913 by F. Stonor of Southampton (Everwilde Farms Inc, 2019). Due to its popularity, Moneymaker has been frequently used as a recurrent parent together with relative tomato accessions as donor parents in many backcross populations, to improve traits such as fruit quality or resistance to plant diseases (Voorrips et al., 2000, Finkers et al., 2008, Víquez-Zamora et al., 2014, Barrantes et al., 2016). *S. arcanum* LA385 is a wild species tomato, originally collected on 1956 at San Juan in Peru, approximately at 2,500m above sea level, and maintained by Tomato Genetics Resource Center (http://tgrc.ucdavis.edu/). LA385 is an indeterminate and allogamous self-incompatible type. It produces small green fruits. This high-altitude wild species was chosen in this research because it has been shown to be tolerant to SOT at the vegetative stage (Venema et al., 1999b) but was not studied at the reproductive stage where plants are most sensitive to temperature stress.

### Growth Conditions and Treatments

Tomato seeds were sown in small pots with commercial potting soil (ED73, Einheitserde, Sinntal-Altengronau, Germany) and germinated seedlings grown for 2 weeks in the glasshouse and then transplanted into separate 17-cm pots containing the same soil. The plants were watered twice a week. After 4 weeks, these plants were moved into two climate chambers. The climate chambers’ growing conditions were: 16-/8-h day/night regime (06.00–22.00-h day and 22.00–06.00-h night), 22/20°C day/night temperature, an irradiance of 300 μmol m^−2^ s^−1^ during the day provided by a mixture of 50% mercury (Master HPI-T Plus, 400 W; Philips, Eindhoven, the Netherlands) and sodium vapor high-pressure lamps (SON-T Agro, 400 W; Philips), 400 ppm CO_2_, and an average relative humidity of 70%. This will be referred to as “control temperature” (CT). The plants were acclimated to the CT for 1 week. From week 5 after sowing, the temperature regime of one climate chamber was changed to 16/14°C day/night while the other parameters remained the same as in CT chamber, and this will be referred to as “SOT.” This SOT regime was chosen because it is below the current optimum temperature 19–20°C for heated glasshouse tomato cultivation in the Netherlands, and well above the non-freezing temperature of 12°C which is known to cause physiological injury to tomato plants and their fruits when exposed for several weeks (Brüggemann et al., 1992, Heuvelink and Dorais, 2005). Plants were given nutrient solution A (1:100 dilution, 20–5–10–2 N–P–K–Mg; Hakaphos, Scotts, OH) weekly during the first 5 weeks, and three times per week from the sixth week onward with nutrient solution B (**Supplementary Table 1**).

This study consisted of two trials. Trial A was from the onset of flowering phase until the young fruit stage, which lasted for 9 weeks from mid-January until the end of March 2015. Trial B was from the stages of young to mature fruit development, which lasted for 16 weeks from beginning of October 2014 until the end of January 2015. Moneymaker is self-compatible, so the pollination of its flowers was done by gently tapping on the base of fluorescence. Meanwhile, *S. arcanum* LA385 is self-incompatible; the plants in trial B were sib pollinated when 75 days old in CT (week 11) and at 68 days old (week 10) in SOT.

### Analysis of Growth and Development

Total leaf area, number of fruits, fresh weight, and dry weight of leaves, stems, fruits, and roots were measured every 2 weeks. Total leaf area was measured by a leaf-area meter (model 3100, LI-COR Inc., Lincoln, NE, USA). Relative leaf thickness was calculated as the division of the total leaf area by leaf fresh weight (Medek et al., 2007). After fresh weight was measured, a small portion of each tissue was collected and snap-frozen in liquid nitrogen and stored at −80°C for further measurements of soluble and phosphorylated sugars and starch contents. The harvested leaves, stems, fruits, and roots were oven-dried at 105°C for 24 h, and then the dry weight was measured.

### CO_2_ Exchange and Chlorophyll Fluorescence Measurement

CO_2_ exchange and chlorophyll fluorescence were measured with an open gas-exchange system Li-Cor 6400 (Li-Cor Inc., Lincoln, NE, USA) equipped with an integrated 2 cm^2^ fluorescence chamber head (Li-6400-40). All measurements were made on the youngest fully expanded leaf after the plants have been in the light for at least 3 h, and between 09.00 and 17.00h. The response of assimilation to irradiance was measured at 400 ppm CO_2_, a gas flow rate of 400 mol s^−1^, a leaf temperature of 22°C (CT) or 16°C (SOT), and a range of irradiances starting from zero up to a saturating irradiance (in some cases an irradiance of 2,000 μmol m^−2^ s^−1^). In addition, two chlorophyll fluorescence–based parameters for each leaf were measured. The maximum quantum efficiency of photosystem II (PSII) photochemistry (Fv/Fm) was measured for each leaf, and the PSII-operating efficiency (Φ_PSII_) was measured for each leaf at each irradiance step. These parameters were calculated using equations 1 and 2:

Equation 1 Fv/Fm=(Fm−Fo)/Fm

where Fv, Fm, and Fo are the variable (i.e., Fm–Fo), maximum, and minimum fluorescence yields measured from 20-min dark-adapted leaf;

Equation 2 ΦPSII=(Fm'−Fs)/Fm'

where Fm’ and Fs are the maximum and steady state fluorescence yields of light-adapted leaves (Baker et al., 2007).

### Soluble Sugars and Starch Content Measurement

Soluble sugars and starch content were measured on the youngest mature leaves and fruits. The extraction of soluble sugars and starch was adapted from the protocol of Kortstee et al. (2007) with some modifications. Twenty milligrams of freeze-dried sample were extracted three times for 40 min with 1 ml of 80% ethanol at 80°C. After each extraction, insoluble material was pelleted by centrifugation (10,000 x *g* for 10 min), and the supernatant was transferred to a new tube. The pellet was kept for starch content measurement. The protocol of soluble sugar content measurement was performed essentially described in Rashidi and Trindade (2018). Five hundred microliters of the supernatant were dried by vacuum centrifugation with a RapidVap (Labconco, Kansas city, MO, USA) and subsequently re-suspended in 500 μl of Milli-Q water (Merck, USA). The clear supernatant was used to measure soluble sugars using HPAEC-PAD Dionex ICS5000+ DC equipped with a Dionex CarboPac PA1 Column (2 x 250 mm) preceded by a similar guard column (2 x 50 mm). The flow rate was 0.25 ml per min, and 2.5 µl of sample were injected using a Dionex AS-AP autosampler. The elution program consisted of an isocratic elution of 10 mM sodium hydroxide in 25min. Each run was followed by a 5-min wash with 1 M sodium acetate in 100 mM sodium hydroxide and a 30-min equilibration with 10 mM sodium hydroxide prior to the next injection. The eluent was monitored by a thermostatic Thermo Scientific ICS5000 pulsed electrochemical detector (PAD). The samples were cooled to 5°C; the Dionex ICS5000+DP column oven temperature was set at 30°C. A series of standard neutral sugars with known concentrations was also run and used for the calculation. The output was subsequently processed with software ChromeleonTM Chromatography Data System version 7 (Thermo Scientific, USA).

Starch content from leaves and fruits was determined using a starch assay kit (no. 0207748, Boehringer, Mannheim, Germany). Briefly, the pellet was solubilized in a solution containing 0.5 ml of 8M HCl and 2 ml of DMSO and then incubated at 60°C for 1 h. The mixture was subsequently neutralized with 0.5 ml of 5 M NaOH and 7 ml of 0.1 M citrate buffer pH 4.6. The insoluble material was pelleted by centrifugation (10,000 x *g* for 1 min). Ten microliters of the clear supernatant was used to determine the starch content.

### Measurement of Phosphorylated Sugars

Phosphorylated sugars were extracted from 30 mg of freeze-dried leaves using the protocol of De Bruijn et al. (1999) with some modifications. Glucose-1,6-bisphosphate was used as the internal standard for phosphorylated sugars. All the standard phosphorylated sugars used in this study, i.e., sucrose-6-phosphate (Suc-6-P), glucose-1-phosphate (Gluc-1-P), glucose-6-phosphate (Glu-6-P), mannose-6-phosphate (Man-6-6P), and fructose-6-phosphate (Fru-6-P), were purchased from Sigma (USA). HPAEC-PAD was used to determine the phosphorylated sugar content and was performed on a Dionex ICS5000+ DC equipped with a Dionex CarboPac PA1 column (2 x 250 mm) proceeded by a similar guard column (2 x 50 mm). The flow rate was 0.25 ml min^−1^, and 5 µl of sample was injected using a Dionex AS-AP autosampler. The elution program consisted of a gradient of sodium acetate from 50 to 800 mM in 40 min in an isocratic background of 4 mM sodium hydroxide. Each run was followed by a 5 min wash with 800 mM sodium acetate and a 15 min equilibration with 50 mM sodium acetate, both in 4 mM sodium hydroxide, prior to the next injection. The eluent was monitored by a temperature controlled Thermo Scientific ICS5000 PAD. The samples were cooled to 5°C, and the Dionex ICS5000+DP column oven was cooled to 10°C. A series of standard neutral sugars with known concentrations was run through the HPLC/detector system to calibrate the assays. The output was subsequently processed with the same software as used in the neutral sugar measurements above.

### Statistical Analysis

One-way analysis of variance (ANOVA) was used to evaluate statistically significant effect of temperature on different parameters aforementioned. We also used two-way ANOVA to evaluate the combined effects of the genotypes and growth temperatures on those same parameters. Each parameter was measured in three to five individual plants. Statistical analyses were carried out with GenStat 17^th^ edition (VSN International Ltd., UK).

## Results

### Plant Growth and Dry Matter Allocation Are Affected by Suboptimal Temperature

**Figure 1:**
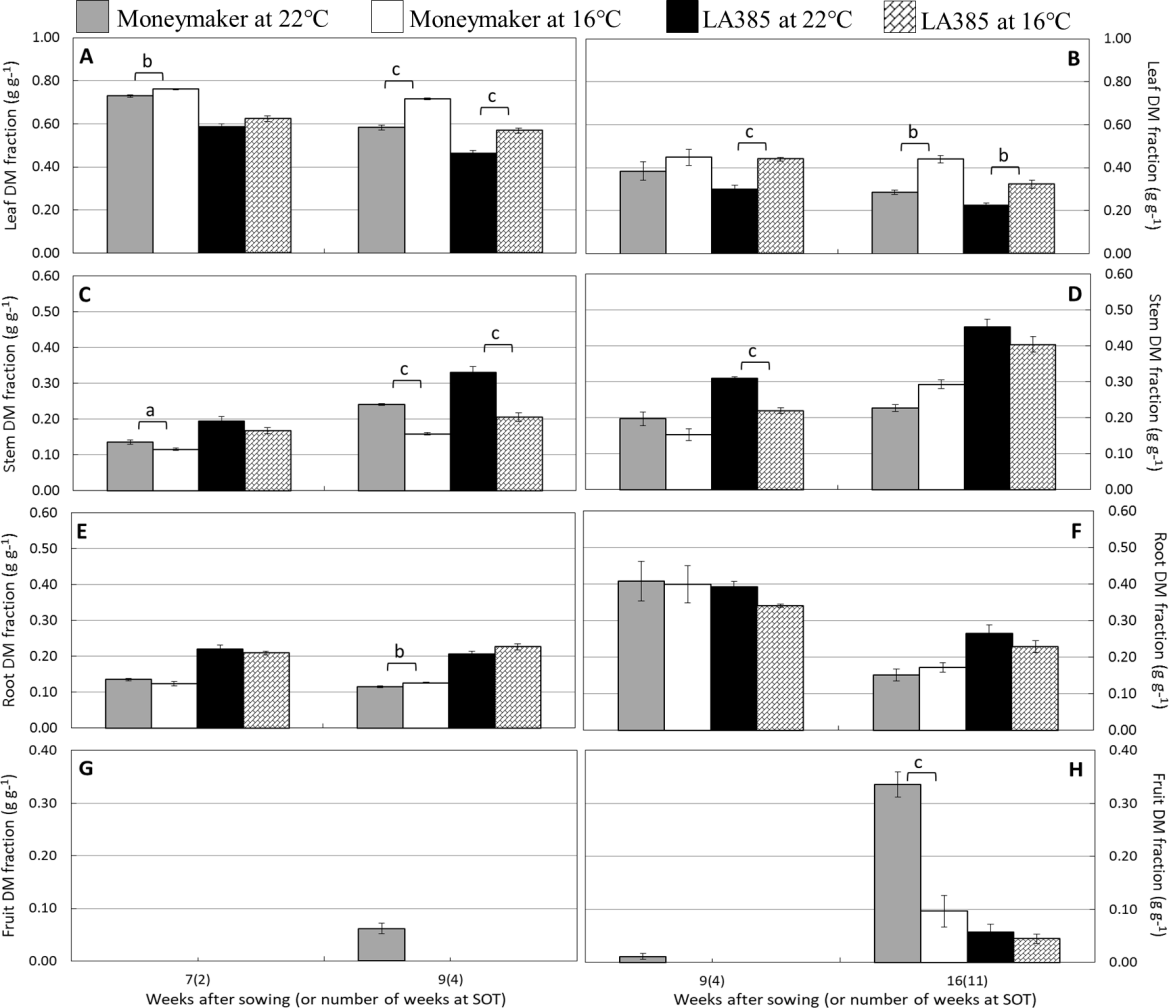
Effect of SOT on dry matter allocation to different tomato organs of cv. Moneymaker and wild accession LA385. DM allocation to leaf, stem, root, and fruit are depicted in **(**panel **A** and **B)**, **(**panel **C** and **D)**, **(**panel **E** and **F)**, and **(**panel **G** and **H)**, respectively. Panels on the left and right are from growth trial **(A)** and trial **(B)**, respectively. The error bars represent standard error of fie individual plants (± SE), except week 9 of trial **(B)** with three plants. Significant difference is denoted as a, b, and c for P < 0.05, < 0.01, and < 0.001, respectively.

Suboptimal temperature strongly affected plant growth and dry matter (DM) allocation in the tomato cultivar Moneymaker, but less so in wild accession LA385 (**Table 1** and **Table 2**). The total leaf area (TLA), specific leaf area (SLA), total fresh weight, total DM, and number of fruits in Moneymaker were significantly reduced at SOT compared to those at CT. In addition, the first fruit harvest in Moneymaker was delayed in SOT (**Table 2**), and in week 16, its fruits were still immature and green in color, while by this stage, there were several ripe, red fruits in CT (data not shown). Furthermore, LA385 showed an acclimation of several parameters to SOT, compared to CT. There was an increase in SLA during the later stages of fruit development, a 1.4-fold higher TLA, accompanied by a two-fold increase in fruit number, a slight decrease in leaf DM content in week 16, and stable total DM yield (**Table 2**). At SOT, both genotypes had thicker leaves and contained slightly more DM in the leaves than in the roots, which was evident from the higher leaf to root ratio (**Table 1** and **Table 2**, and see also **Supplementary Figure 1**). Furthermore, DM allocation to different plant organs was also affected at SOT (**Figure 1**). In both genotypes, more DM was allocated to leaves (**Figures 1A,B**), and less DM was partitioned to stems (**Figures 1C, D**) at SOT than at CT. The fraction of DM allocation to roots was not significantly affected by temperature (**Figures 1E, F**). The DM allocation to fruits in Moneymaker dropped significantly from 34% at SOT to 10% at CT in week 16, whereas it remained stable at around 5% in LA385 (**Figures 1G, H**). The interactive effect of temperature and genotypes in many of these parameters were also demonstrated in **Supplementary Table 2**.

**Table 1:** Growth parafmeters of *Solanum lycopersicum* cv. Moneymaker and wild species *Solanum arcanum* LA385 at control (22°C) and suboptimal temperatures (16°C) in trial A.

	T(°C)	Moneymaker		LA385	
		Week 7 (2)*	Week 9 (4)	Week7 (2)	Week 9 (4)
Total leaf area (cm^2^)	22	2026 ± 48	3467 ± 84	714 ± 3	1191 ± 40
	16	2168 ± 48	3027 ± 101 a	741 ± 14	1083 ± 29
Specific leaf area (cm^2^ g DW ^−1^)	22	181.3 ± 4.8	150.9 ± 3.5	198.6 ± 3.8	135.3 ± 6.8
	16	170.4 ± 2.8	119.6 ± 2.0 c	170.6 ± 3.8 c	104.4 ± 3.5 b
Relative leaf thickness	22	0.051 ± 0.002	0.051 ± 0.001	0.063 ± 0.009	0.043 ± 0.001
	16	0.048 ± 0.000	0.058 ± 0.001 b	0.042 ± 0.001 a	0.049 ± 0.004
Total fresh weight (g plant^−1^)	22	150.5 ± 6.0	322.5 ± 5.2	67.9 ± 8.0	120.8 ± 6.1
	16	144.6 ± 5.8	252.9 ± 4.9 c	57.1 ± 2.1	102.7 ± 5.8
Total dry matter (g plant^−1^)	22	15.3 ± 0.4	39.4 ± 0.5	5.9 ± 0.2	19.3 ± 1.5
	16	16.8 ± 0.7	35.3 ± 0.8 b	7.3 ± 0.3 b	18.4 ± 1.2
Leaf dry matter content (%)	22	11.0 ± 0.7	13.1 ± 0.1	8.8 ± 1.5	17.4 ± 0.9
	16	12.3 ± 0.1	14.5 ± 0.2 c	13.9 ± 0.1 a	20.0 ± 1.1
Leaf to root dry mater ratio (g g^−1^)	22	5.43 ± 0.16	5.13 ± 0.18	2.71 ± 0.17	2.26 ± 0.10
	16	6.24 ± 0.34	5.72 ± 0.12 a	3.01 ± 0.14	2.54 ± 0.13
Number of fruits	22	n.a.	10 ± 0	n.a.	6 ± 1
	16	n.a.	n.a.	n.a.	n.a.

* (n), number of weeks exposed to SOT; n.a., not available. Data represent the mean of five individual plants ( ± SE). Significant differences are denoted as a, b, and c for P < 0.05, < 0.01, and < 0.001, respectively.

**Table 2:** Growth parameters of *Solanum lycopersicum* cv. Moneymaker and wild species *Solanum arcanum* LA385 at control (22°C) and suboptimal temperatures (16°C) in trial B.

	T (°C)	Moneymaker		LA385	
		Week 9 (4)*	Week 16 (11)	Week 9 (4)	Week 16 (11)
Total leaf area (cm^2^)	22	2427 ± 48	5996 ± 451	711 ± 57	1568 ± 99
	16	1682 ± 60 c	4345 ± 328 a	707 ± 48	2149 ± 123 b
Specific leaf area (cm^2^ g DW^−1^)	22	107.0 ± 1.0	163.6 ± 6.3	110.1 ± 2.4	126.9 ± 2.7
	16	89.5 ± 3.4 b	118.9 ± 9.2 b	86.5 ± 4.4 b	123.8 ± 1.8
Relative leaf thickness	22	0.052 ± 0.001	0.051 ± 0.004	0.040 ± 0.000	0.044 ± 0.002
	16	0.055 ± 0.001	0.067 ± 0.006	0.049 ± 0.001 b	0.051 ± 0.001 b
Total fresh weight (g plant^−1^)	22	343.0 ± 25.7	1284.6 ± 64.9	132.9 ± 2.3	325.2 ± 14.7
	16	240.4 ± 8.7 a	644.8 ± 25.2 c	103.9 ± 3.4 b	325.4 ± 14.4
Total dry matter (g plant^−1^)	22	60.2 ± 5.7	128.0 ± 7.1	21.5 ± 0.6	54.5 ± 2.7
	16	42.5 ± 3.5	83.6 ± 3.5 c	18.6 ± 1.3	54.0 ± 2.5
Leaf dry matter content (%)	22	17.9 ± 0.1	12.2 ± 0.4	22.6 ± 0.7	18.1 ± 1.0
	16	20.5 ± 1.1	13.1 ± 0.8	23.6 ± 0.6	15.9 ± 0.3
Leaf to root dry mater ratio (g g^−1^)	22	1.02 ± 0.27	1.98 ± 0.19	0.77 ± 0.08	0.88 ± 0.07
	16	1.18 ± 0.23	2.60 ± 0.11 a	1.30 ± 0.04 b	1.44 ± 0.12 b
Number of fruits	22	6 ± 1	26 ± 2	n.a.	17 ± 4
	16	n.a.	18 ± 2 a	n.a.	30 ± 2 a

* (n), number of weeks exposed to SOT; n.a., not available. Data represent the mean of three individual plants in week 9 and five individual plants in week 16 ( ± SE). Significant differences are indicated with a, b, and c for P < 0.05, < 0.01, and < 0.001, respectively.

### Chlorophyll Fluorescence Parameters and CO_2_ Assimilation Are Affected by SOT in cv. Moneymaker but to a Lower Extent in Wild Accession LA385

The maximum quantum efficiency of PSII photochemistry (F_v_/F_m_) in both genotypes was not significantly affected at SOT, while the PSII-operating efficiency (Φ_PSII_) (measured at 300 μmol m^−2^ s^−1^) was affected (**Table 3** and **Supplementary Figure 2**). In the case of Moneymaker, Φ_PSII_ was significantly decreased at SOT compared to CT, with reductions of 26 and 18% at week 7 and week 16, respectively. In LA385, Φ_PSII_ also decreased at SOT compared to CT, but only by 2–5%. Furthermore, we also observed a positive correlation between Φ_PSII_ and net CO_2_ assimilation rate (A_N_) (**Table 3** and **Supplementary Figure 3**). As with Φ_PSII_, the A_N_ (measured at 300 μmol m^−2^ s^−1^) of both genotypes were affected by SOT compared to CT, but the impact was less significant for LA385 than for Moneymaker, especially at week 16. In addition, the light-saturated CO_2_ assimilation rate (A_sat_) of Moneymaker was much lower at SOT in both weeks 7 and 16, whereas A_sat_ of LA385 was only slightly affected in SOT compared to CT. The combined effect of temperature and genotype was most clear in A_N_ at week 7 and in Φ_PSII_ at both weeks 7 and 16 (**Supplementary Table 2**).

**Table 3:** Influence of SOT on Fv/Fm, net CO_2_ assimilation rate (A_N_) and Φ_PSII_ at PFD of 300 µmol m^−^^2^ s^−^^1^, and light-saturated CO_2_ assimilation rate (A_sat_).

	T (°C)	Moneymaker		LA385	
		Week 7 (2)*	Week 16 (11)	Week 7 (2)	Week 16 (11)
Fv/Fm	22	0.810 ± 0.001	0.806 ± 0.006	0.820 ± 0.003	0.816 ± 0.006
	16	0.804 ± 0.001 b	0.811 ± 0.002	0.822 ± 0.002	0.817 ± 0.008
Φ_PSII_	22	0.613 ± 0.012	0.573 ± 0.020	0.675 ± 0.006	0.651 ± 0.013
	16	0.452 ± 0.016 b	0.467 ± 0.024 a	0.644 ± 0.007 a	0.639 ± 0.013
A_N_	22	10.9 ± 0.2	14.6 ± 0.9	13.1 ± 0.2	14.8 ± 1.6
	16	3.4 ± 0.4 c	8.3 ± 0.4 c	8.5 ± 0.2 c	11.7 ± 1.1
A_sat_	22	15.5 ± 0.4	21.6 ± 1.4	28.9 ± 2.1	24.9 ± 2.6
	16	7.0 ± 0.3 c	12.8 ± 0.5 c	23.6 ± 1.4	20.1 ± 2.0

Data represent the mean ( ± SE) of three to five individual plants. F_v_/F_m_ and *Φ*_PSII_ are dimensionless, while the units of A_N_ and A_sat_ are µmol m^−2^ s^−1^. Significant difference is denoted as a, b, and c for P < 0.05, < 0.01, and < 0.001, respectively. *(n) is number of weeks exposed to SOT.

### High Amount of Sucrose-6-Phosphate Was Detected in Leaves of the Wild Accession LA385

**Figure 2:**
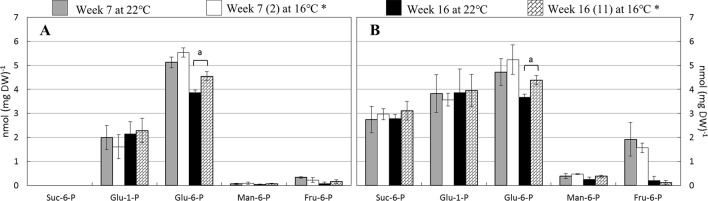
Effect of SOT on several phosphorylated sugars in leaves at weeks 7 and 16. **(**Panel **A** and **B)** depicts sucrose-6-phosphate (Suc-6-P), glucose-1-phosphate (Gluc-1-P), glucose-6-phosphate (Glu-6-P), manose-6-phosphate (Man-6-6P), and fructose-6-phosphate (Fru-6-P) content of cv. Moneymaker and wild accession LA385, respectively. (n) is the number of weeks exposed to SOT. The error bars represent standard error of mean of five plants. Significant difference is denoted as “a” for P < 0.05.

Five distinct, phosphorylated sugars were measured in the leaf samples, i.e., sucrose-6-phosphate (Suc-6-P), glucose-1-phosphate (Glu-1-P), glucose-6-phosphate (Glu-6-P), mannose-6-phosphate (Man-6-P), and fructose-6-phosphate (Fru-6-P) (**Figure 2**). Strikingly, Suc-6-P was abundant in LA385 leaves but was not detected to any significant level in Moneymaker leaves. Furthermore, the content of Man-6-P and Fru-6-P was also higher in LA385 than in Moneymaker (**Supplementary Table 2**). In general, the phosphorylated sugar profile in leaves of both genotypes was not affected at SOT, except Glu-6-P content in week 16, which was higher at 16°C compared to 22°C. In addition, it appeared that there was no combined effect of temperature and genotype on the contents of these phosphorylated sugars (**Supplementary Table 2**).

### Cultivar Moneymaker and Wild Accession LA385 Had Different Carbohydrate Profiles in Fruits and Leaves

**Figure 3:**
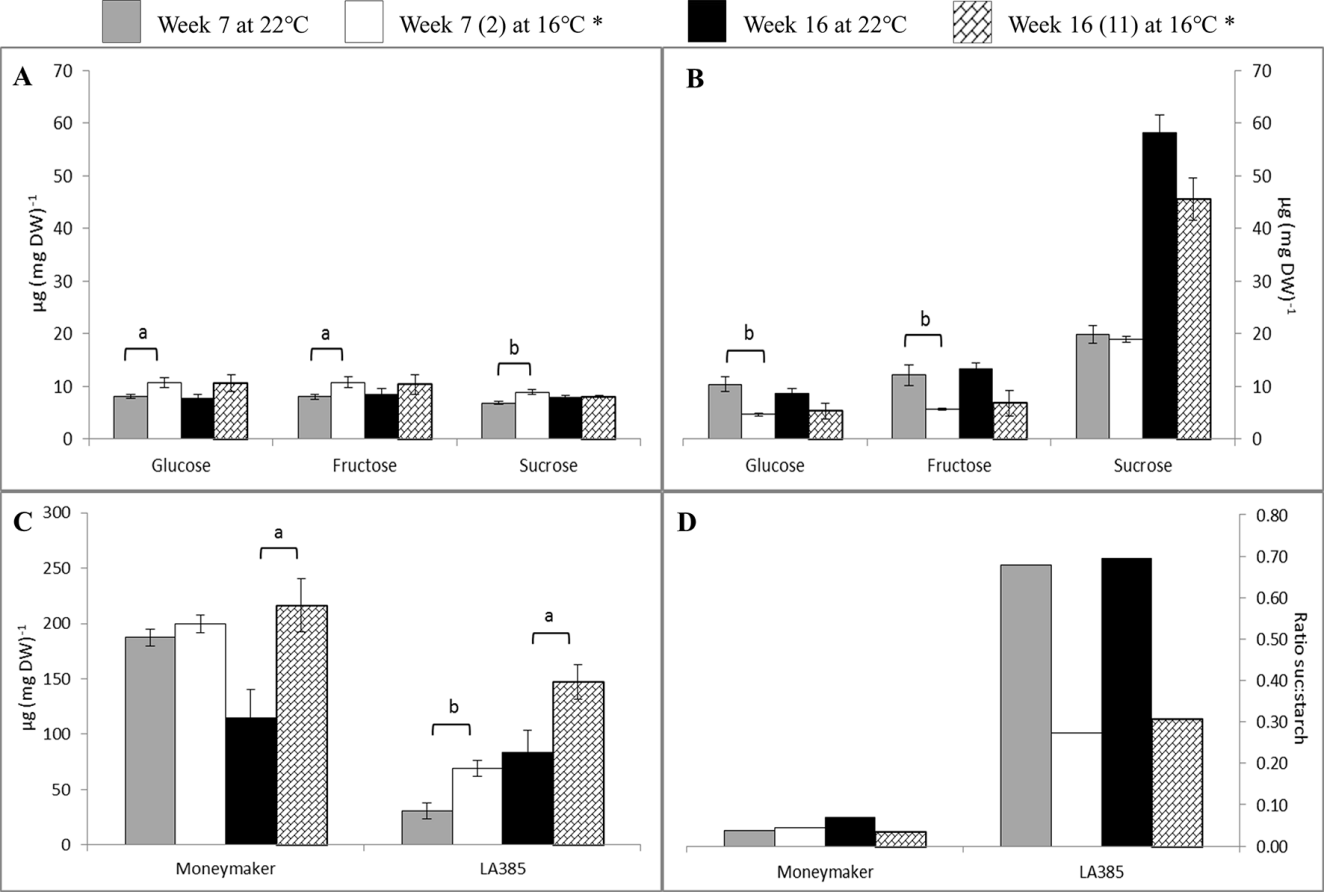
Effect of SOT on neutral sugars, starch content, and sucrose: starch ratio in leaves. **(**Panels **A** and **B)** show the glucose, fructose, and sucrose contents at weeks 7 and 16 of cv. Moneymaker and wild accession LA385, respectively. **(**Panels **C** and **D)** show the starch content and sucrose: starch ratio, respectively. *(n) is the number of weeks exposed to SOT. The error bars represent standard error of mean of five plants. Significant difference is denoted as a, b, and c for P < 0.05, < 0.01, and < 0.001, respectively.

The neutral sugars and starch profiles in leaves of Moneymaker and LA385 (**Figure 3**) were different. Moneymaker leaves accumulated significantly more glucose, fructose, and sucrose at SOT than at CT in week 7, but this accumulation of neutral sugars stopped in week 16 (**Figure 3A**). Compared to Moneymaker, LA385 leaves displayed an opposite trend of neutral sugar accumulation: they contained fewer neutral sugars at SOT, especially fructose and glucose but accumulated more sucrose (**Figure 3B**). As the LA385 plants became more mature, the sucrose content of their leaves increased more than two-fold (cf week 7 leaves with week 16 leaves). This interactive effect of temperature and genotype on sucrose content in leaves was also shown in **Supplementary Table 2**. Another striking difference between Moneymaker and LA385 leaves was their starch content (**Figure 3C**). Compared to LA385, Moneymaker leaves had a high-starch content of approximately 200 µg mg DW^−1^ in leaves at both temperatures in week 7, which decreased by about 50% at CT by week 16 while the starch content of the SOT leaves at week 16 similar to the levels found at week 7. The starch content of the LA385 leaves increased both with age, being higher at week 16 than week 7, and with decreased growth temperature, being higher at SOT than at CT. These effects of age and growth temperature for LA385 leaves were approximately additive, so the lowest starch levels were found in CT leaves at week 7, and the highest at SOT leaves at week 16. Though starch content in LA385 was higher at week 16 than week 7; it was at week 7 that fruits had begun to form and fruits are strong sinks. Overall, the ratio of sucrose to starch content in Moneymaker leaves was approximately 4 and 7% at SOT and CT, respectively. Compared to Moneymaker leaves, the ratio of sucrose over starch content in LA385 leaves were about 10-fold higher, i.e., 30 and 70% at SOT and CT, respectively (**Figure 3D**).

**Figure 4:**
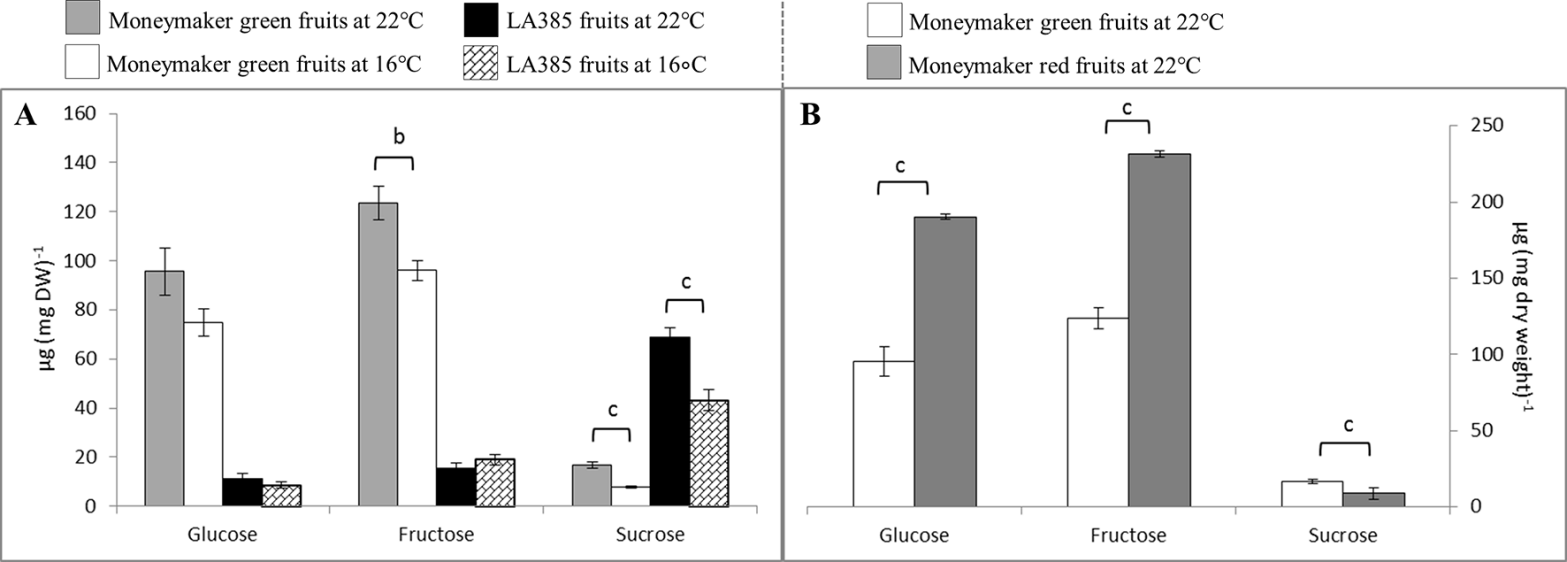
Effect of SOT on neutral sugars in fruits of cv. Moneymaker (MM) and wild accession LA385 at week 16 (or 11 weeks exposed to SOT). **(**Panel **A)** displays the neutral sugar profile in green fruits of both tomato genotypes, while **(**Panel **B)** shows the content of neutral sugars in green and red fruits of cv. Moneymaker. The error bars represent standard error of mean of five different plants. Significant difference is denoted as a, b, and c for P < 0.05, < 0.01, and < 0.001, respectively.

The profile of neutral sugars in fruits was distinct between two genotypes. Hexoses (i.e., glucose and fructose) and sucrose are the primary sugars of Moneymaker and LA385, respectively (**Figure 4**). Moneymaker immature green fruits contained 13-fold and 22-fold more hexoses compared to sucrose at CT and SOT, respectively; while its ripe red fruits contained two-fold more hexoses and two-fold less sucrose compared to the green fruits at CT (**Figure 4B**). On the other hand, the predominant neutral sugar in LA385 fruits was sucrose which was at least two-fold higher than hexoses (**Figure 4A**). The neutral sugar content of Moneymaker and LA385 fruits appeared to be affected differently at SOT. The content of glucose, fructose, and sucrose in Moneymaker fruits was sharply decreased at SOT, whereas only the sucrose content of LA385 fruits was significantly reduced at SOT. This interactive effect of temperature and genotype on neutral sugars was also displayed in **Supplementary Table 2**, especially for fructose and sucrose.

## Discussion

### Wild Accession LA385 Has Better Acclimation From Affected Plant Morphology at Suboptimal Temperature

Suboptimal temperature (SOT) treatment resulted in a clear delay in growth and development in the *S. lycopersicum* cv. Moneymaker, whereas the high-altitude wild species S. *arcanum* LA385 was able to acclimate from the SOT-induced delay during the fruit development phase. The reduction in SLA at SOT in Moneymaker (**Table 1**) is in agreement with previous reports (Venema et al., 1999b, Atkin et al., 2006, Kläring et al., 2015). Given that the leaf DM content in Moneymaker was unaffected by SOT (**Supplementary Figures 1A, B**), the reduction of SLA was likely offset by the increase in leaf thickness. According to Hoek et al. (1993), the larger size of the cells at SOT is the cause of the increase in leaf thickness, and these larger cells are able to store more starch (Venema et al., 1999a, Klopotek and Kläring, 2014). In addition, the increase in leaf thickness and total protein concentration have been suggested to be connected to acclimation to low temperature (Pyl et al., 2012). In this study, we observed that, at early growth stage, the SLA of LA385 was reduced at SOT, which is in line with the findings of Venema et al. (1999b) (who reported only on young tomato plants). Furthermore, we show, for the first time, that the SLA of LA385 at SOT was able to acclimate to close to the value at CT during the fruit development stage. It is possible that this acclimation of SLA at SOT was due to the slight reduction in leaf DM content of wild accession LA385 in week 16 (**Supplementary Figure 1A**). In turn, the slight decrease in leaf DM content of LA385 at SOT had a smaller effect on the DM allocation to other plant organs and helped maintain whole plant growth.

### Suboptimal Temperature Decreases Dry Matter Yield and Fruit Growth in cv. Moneymaker but Not in the Wild Accession LA385

Suboptimal temperature strongly affected the total DM production and DM allocation to fruits in cv. Moneymaker, whereas it had a negligible influence on those parameters in the high-altitude wild accession LA385 (**Table 1**, **Table 2**, and **Supplementary Table 2**). At both CT and SOT conditions, however, DM allocation to fruits in LA385 was always lower than DM allocation to fruits in Moneymaker. The drop in total DM yield of Moneymaker at SOT was paralleled by the sharp decrease in its DM allocation to fruits (**Table 1**, **Table 2** and **Figure 1**). These reductions could be attributed to the profoundly lower accumulation of neutral sugars in the green fruits of Moneymaker at SOT in contrast to that found in ripe fruits at CT (**Figure 4B**). Furthermore, this study also observed the delay of first fruit harvest in cultivated tomatoes due to lower temperatures, as reported by others (Hurd and Graves, 1985, Kläring et al., 2015); there were no ripe fruits on Moneymaker at SOT in week 16, while by week 16, several ripe fruits had developed at CT (data not shown). Therefore it is likely that, compared to CT, a longer growing time at SOT would be required for fruit ripening in Moneymaker and for the accumulation of DM to the levels found at CT. This hypothesis is supported by previous research in which the tomato cultivars grown for at least 4 weeks longer at SOT than in this study showed no reduction in the total DM yield and DM fraction to fruits at SOT compared to CT (Heuvelink, 1995, Kläring et al., 2015). We also show here, for the first time, the impact of low temperatures on the total DM yield and DM allocation of LA385 from flower onset until fruit development stage. The stability of total DM yield and DM fraction to sink fruits at SOT compared to CT demonstrates the cold tolerance of this wild accession and its potential value as a source of this trait. The cold tolerance of LA385 is likely due to its ability to acclimate its source-sink balance during fruit development stage, which could be seen in the pronounced increase in TLA and number of sink fruits and similar level of DM allocation to fruits of LA385 at SOT compared to CT (**Table 1**, **Table 2**, and **Figure 1**)—the ability of LA385 to continue to grow better at SOT than Moneymaker plays a role in this.

### Suboptimal Temperature Strongly Affects Photosynthetic Capacity of Cultivated Tomato but Less in Wild Accession

Photosynthesis in LA385 shows much better adaptation to SOT than does that of Moneymaker. The maximum, dark-adapted quantum efficiency of PSII photochemistry, which is estimated as the ratio of variable chlorophyll fluorescence yield and the maximum fluorescence yield (F_v_/F_m_), is known to be a good parameter with which to measure the accumulation of stress in a plant. The F_v_/F_m_ of C3 plants under optimal growth condition is around 0.83 (when measured with red excitation of PSII), and a value below 0.80 is generally taken to indicate the advent of photoinhibition (Björkman and Demmig, 1987). All the F_v_/F_m_ of both plant genotypes in this study were around 0.81–0.82 despite of the low temperatures to which some plants were exposed (**Table 3**). Therefore, the plants in this study did not experience a temperature stress to an extent necessary to produce damage to PSII. However, SOT had different effects on the CO_2_ assimilation rates of Moneymaker and LA385. In the case of Moneymaker at SOT, we observed a significant reduction in both the leaf net CO_2_ assimilation rate (A_N_) at growth irradiance (300 μmol m^−2^ s^−1^) and the light-saturated assimilation rate (A_sat_) (**Table 3** and **Supplementary Figure 2**). This reduction in A_N_ was paralleled by a decreased Φ_PSII_ under these conditions at SOT (**Table 3**). It was clear that, at SOT, Moneymaker was not able to sustain its photosynthetic capacity at levels comparable to those found at CT. In contrast to Moneymaker, we report for the first time that the wild accession LA385 could acclimate well to SOT; its CO_2_ assimilation rates and Φ_PSII_ at SOT were similar to those found at CT in week 16, during the fruit development phase (**Supplementary Table 2**). Additionally, all photosynthetic properties measured, i.e., Fv/Fm, Φ_PSII_, A_N_, and A_sat_, were consistently higher in LA385 than in Moneymaker throughout development and at both temperatures (**Table 3** and **Supplementary Figure 2**). These findings indicate that LA385 is a potential source for the improvement of photosynthetic capacity in cultivated tomatoes.

### Higher Sucrose Metabolism Plays an Important Role in Photosynthetic Capacity

Downstream of photosynthesis is carbohydrate metabolism, in which fixed carbon is converted to end products, such as sucrose and starch, which can be used for plant growth and storage (reviewed in Smith and Stitt, 2007). With higher photosynthetic capacity than Moneymaker, leaves of LA385 also appeared to have differential regulation or limitation of sucrose biosynthesis than Moneymaker (**Supplementary Table 2**). This could be seen *via* the differences in suc-6-P, a precursor of sucrose, and sucrose contents in leaves of these two distinct tomato accessions: (i) while suc-6-P was abundant in LA385, it could not be detected in Moneymaker (**Figure 2**), and (ii) LA385 leaves also had twice the sucrose content of Moneymaker leaves at week 7 and five times the sucrose content at week 16 (**Figures 3A, B**). In plants, synthesis of sucrose is governed by two enzymes: sucrose phosphate synthase (SPS) catalyzes the synthesis of suc-6-P from fructose-6-phosphate (Fru-6-P) and UDP-glucose (Stitt et al., 1988); and sucrose phosphate phosphatase (SPP) irreversibly hydrolyzes suc-6-P into sucrose. *Via* isotope dilution experiments, SPS and SPP have been suggested to form a complex facilitating the process of sucrose synthesis from suc-6-P (Echeverria et al., 1997). Using techniques such as bioluminescence resonance energy transfer (BRET) and bimolecular fluorescence complementation (BiFC), Maloney et al. (2015) confirmed that SPS and SPP indeed interact and form complex *in planta*, and overexpression of this complex in Arabidopsis and poplar promotes plant growth. Therefore, it is possible that LA385 has a more efficient SPS-SPP complex than Moneymaker, which needs to be further investigated. On the other hand, no clear connection between the accumulation of soluble sugars or phosphorylated sugars in plant leaves and the decrease in photosynthetic capacity could be established in this study. Additionally, the fruits of LA385 accumulated four- to five-fold as much sucrose as counterparts of Moneymaker (**Figure 4**), which is in agreement with other work that showed LA385 is a sucrose accumulator and cultivated tomatoes such as cv. Moneymaker is a hexose accumulator (Kortstee et al., 2007). Based on the osmotic principle, the accumulation of disaccharides (i.e., sucrose) instead of monosaccharides (i.e., hexoses) in sink organs would diminish the water uptake, which would then result in sinks with higher soluble solid content but lesser mass. The effect of tomato fruit sucrose accumulation was investigated by Chetelat et al. (1995), in which the authors generated BC5F2 plants containing the gene controlling fruit sucrose accumulation by backcrossing a high sucrose accumulator wild relative *Solanum chmielewskii* LA1028 with a cultivated tomato background as a hexose accumulator. The authors showed that there was no difference in total fruit mass between the sucrose and hexose accumulators, but sucrose accumulating plants were associated with several fruit quality parameters such as higher soluble solid content, juice consistency, serum viscosity, and predicted paste yield. However, those authors did not include photosynthetic capacity measurement in their work. Therefore, further study needs to be done to investigate the effect of high sucrose accumulation in fruits on photosynthetic capacity. In addition to sugar metabolism, starch metabolism is another key process downstream of photosynthesis responsible for formation, breakdown, and interconversion of carbohydrate in plants. Both genotypes in our study showed significant accumulation of starch in their leaves at SOT, except for Moneymaker in week 7 (**Figure 3C**). In those plants with significantly higher accumulation of starch in leaves at SOT, it appeared that their photosynthetic parameters, such as Φ_PSII_, A_N_, and A_sat_, were lower than those at CT (**Figure 3C** and **Table 3**). However, Moneymaker plants at SOT in week 7 having similar leaf starch content as the counterparts at CT also had those photosynthetic parameters significantly reduced at SOT compared to CT. Therefore, leaf starch content did not seem to associate with the photosynthetic capacity. Comparing two cultivars and two high-altitude wild species tomato young plants grown at 25/20 and 16/14°C, Venema et al. (1999b) also observed a significantly higher starch accumulation in leaves of all four genotypes at 16/14°C than at 25/20°C, but no major decline in their photosynthetic capacity. In another study, Goldschmidt and Huber (1992) showed a correlation in a reduction in maximum photosynthetic capacity (A_max_) and high leaf starch content among several plant species responded to girdling y*et al*so observed a high percentage of inhibition in A_max_ in girded leaves of a starchless tobacco mutant. Similar findings were also found in the work of Li et al. (2018), in which those authors exposed two zoysiagrass genotypes, cold tolerant one native to cold climate area, and cold sensitive one from warm climate are, to sub-optimal 18/12°C, chilling 8/2°C and freezing 2/−4°C temperatures compared to the control 30/25°C. Those authors observed that the cold tolerant genotype had a slight higher A_N_ and a significantly higher accumulation of sucrose, trehalose, fructan, and starch comparing to the cold sensitive genotype at lower temperatures. Together with these studies, the data of our study also suggests that that the high accumulation of starch is not the main cause for reduction in photosynthesis capacity. Furthermore, there was a clear distinction in sucrose: starch ratio between the leaves of Moneymaker and LA385, in which this ratio in LA385 was 10-fold higher than in Moneymaker (**Figure 3D**). This finding implies that the distinct sucrose metabolism in both leaves and fruits of wild accession LA385 could be one of the keys to support its higher photosynthetic capacity compared to that in Moneymaker. We have shown in a recent study that the heterologously expressed protein sucrose synthase 3 (SUSY3) of LA385 could hydrolyze sucrose more efficiently compared to the counterpart of Moneymaker (Dinh et al., 2018). Additionally, SUSY has been shown to interact with multiprotein complex involved in cellulose/callose biosynthesis in main crops such as cotton, tobacco, and wood (Nakai et al., 1999, Persia et al., 2008, Coleman et al., 2009, Fujii et al., 2010). Interestingly, Ntatsi et al. (2017) reported an increase in expression of genes related to cellulose biosynthesis in the rootstock of cold tolerant tomato *Solanum habrochaites* LA1777 when grown at suboptimal root temperature of 15°C, whereas in the same growth temperature those genes were not up-regulated in the rootstock of cold sensitive tomato Moneymaker. Therefore, SUSY and other players involved in sucrose metabolism would be of interest for further investigation.

## Conclusion

Growth at SOT leads to different effects on the tomato cultivar Moneymaker and the high-altitude wild species *S. arcanum* LA385 with regards their growth, development, DM production and allocation, photosynthetic capacity, and carbohydrate metabolism from the reproductive phase to the later phases of fruit development. To our knowledge, this current work is the first report on the effect of SOT with a wild species during the reproductive stage, where plants are most sensitive to temperature stress, up to the later stage of fruit development. While cv. Moneymaker is profoundly affected by SOT, the wild accession LA385 is shown to acclimate well to SOT at the stage of fruit development with high photosynthetic capacity accompanied by active sucrose metabolism. Among our findings, the highly active sucrose metabolism in wild accession LA385 is the most interesting lead that we will investigate further. Getting more insights about the sucrose metabolism in this wild accession at SOT might facilitate the breeding program for new cultivars performing well at SOT, which might allow reductions in energy use for heating and bring the tomato greenhouse cultivation in temperate region one step closer to sustainability.

## Author Contributions

Q-DD and LT did the experimental planning; Q-DD, AD, HF, and TG performed data acquisition and analysis; Q-DD drafted the manuscript; RV, JH, and LT reviewed the manuscript critically. LT coordinated the project’s funding.

## Funding

This work was carried out within the research programme of BioSolar Cells led by René Klein Lankhorst, co-financed by the Dutch Ministry of Economic Affairs.

## Conflict of Interest Statement

The authors declare that the research was conducted in the absence of any commercial or financial relationships that could be construed as a potential conflict of interest.
