# Supplementary material for: High-Altitude Wild Species Solanum arcanum LA385—A Potential Source for Improvement of Plant Growth and Photosynthetic Performance at Suboptimal Temperatures
Source: Front Plant Sci. 2019 Sep 24;10:1163. doi: 10.3389/fpls.2019.01163 (PMC6769098; doi:10.3389/fpls.2019.01163)
Supplement: Supplementary file 1 [file DataSheet_1.docx]

Supplementary Material

High altitude wild species *Solanum arcanum* LA385 – a potential source for improvement of plant growth and photosynthetic performance at suboptimal temperatures

Quy-Dung Dinh^1,2^, Annemarie Dechesne^1^, Heleen Furrer^1^, Graham Taylor^3^, Richard G. F. Visser^1^, Jeremy Harbinson^3^, and Luisa M. Trindade^1*^

* **Correspondence**: Luisa Trindade: [luisa.trindade@wur.nl](mailto:luisa.trindade@wur.nl)

**Supplementary Table 1.** Concentration of macro- and micro-nutrients inside the nutrient solution B with EC 2.1 and pH 5.5

| **Nutrient** | **mmol l^-1^** | **micromole l^-1^** |
| --- | --- | --- |
| NH_4_ | 1.2 |  |
| K | 7.2 |  |
| Ca | 4.0 |  |
| Mg | 1.82 |  |
| NO_3_ | 12.4 |  |
| SO4 | 3.3 |  |
| P | 1.0 |  |
| Fe |  | 35.0 |
| Mn |  | 8.0 |
| Zn |  | 5.0 |
| B |  | 20.0 |
| Cu |  | 0.5 |
| Mo |  | 0.5 |

**Supplementary Table 2:** *P*-values of a two-way ANOVA testing for the effects of temperature treatment (22°C vs 16°C) and genotypes (cv Moneymaker vs wild species LA385) on all the studied parameters at week 7 (2 weeks at SOT) and week 16 (11 weeks at SOT)

| Parameters | Week | Temperature | Genotype | Temperature * Genotype |
| --- | --- | --- | --- | --- |
| Total leaf area | W7 (2) | < 0.05 | < 0.001 | 0.592 |
|  | W16 (11) | 0.084 | < 0.001 | < 0.01 |
| Specific leaf area | W7 (2) | < 0.001 | < 0.05 | < 0.05 |
|  | W16 (11) | < 0.001 | < 0.05 | < 0.01 |
| Relative leaf thickness | W7 (2) | < 0.05 | 0.469 | 0.063 |
|  | W16 (11) | < 0.01 | < 0.01 | 0.308 |
| Total fresh weight | W7 (2) | 0.175 | < 0.001 | 0.683 |
|  | W16 (11) | < 0.001 | < 0.001 | < 0.001 |
| Total dry weight | W7 (2) | < 0.01 | < 0.001 | 0.981 |
|  | W16 (11) | < 0.001 | < 0.001 | < 0.001 |
| Leaf dry matter content | W7 (2) | < 0.01 | 0.760 | < 0.05 |
|  | W16 (11) | 0.354 | < 0.001 | < 0.05 |
| Leaf to root DM ratio | W7 (2) | < 0.05 | < 0.001 | 0.254 |
|  | W16 (11) | < 0.001 | < 0.001 | 0.816 |
| Number of fruits | W7 (2) | n/a | n/a | n/a |
|  | W16 (11) | 0.343 | 0.644 | < 0.01 |
| Leaf DM fraction | W7 (2) | < 0.01 | < 0.001 | 0.762 |
|  | W16 (11) | < 0.001 | < 0.001 | 0.055 |
| Stem DM fraction | W7 (2) | < 0.05 | < 0.001 | 0.820 |
|  | W16 (11) | 0.570 | < 0.001 | < 0.01 |
| Root DM fraction | W7 (2) | 0.085 | < 0.001 | 0.889 |
|  | W16 (11) | 0.663 | < 0.001 | 0.140 |
| Fruit DM fraction | W7 (2) | n/a | n/a | n/a |
|  | W16 (11) | < 0.001 | < 0.001 | < 0.001 |
|  |  |  |  |  |

**Supplementary Table 2 (cont.):** *P*-values of a two-way ANOVA testing for the effects of temperature treatment (22°C vs 16°C) and genotypes (cv Moneymaker vs wild species LA385) on all the studied parameters at week 7 (2 weeks at SOT) and week 16 (11 weeks at SOT)

| Parameters | Week | Temperature | Genotype | Temperature * Genotype |
| --- | --- | --- | --- | --- |
| Sucrose-6-phosphate in leaf | W7 (2) | 0.709 | < 0.001 | 0.709 |
|  | W16 (11) | 0.447 | < 0.001 | 0.447 |
| Glucose-1-phosphate in leaf | W7 (2) | 0.564 | < 0.01 | 0.902 |
|  | W16 (11) | 0.862 | < 0.05 | 0.973 |
| Glucose-6-phosphate in leaf | W7 (2) | 0.307 | 0.434 | 0.917 |
|  | W16 (11) | < 0.001 | 0.303 | 0.899 |
| Mannose-6-phosphate in leaf | W7 (2) | 0.357 | < 0.001 | 0.613 |
|  | W16 (11) | 0.158 | < 0.001 | 0.358 |
| Fructose-6-phosphate in leaf | W7 (2) | 0.547 | < 0.01 | 0.747 |
|  | W16 (11) | 0.961 | 0.620 | 0.412 |
| Glucose in leaf | W7 (2) | 0.126 | < 0.05 | < 0.001 |
|  | W16 (11) | 0.994 | 0.081 | < 0.05 |
| Fructose in leaf | W7 (2) | 0.452 | 0.283 | < 0.05 |
|  | W16 (11) | 0.248 | 0.870 | < 0.05 |
| Sucrose in leaf | W7 (2) | 0.855 | < 0.001 | < 0.05 |
|  | W16 (11) | < 0.05 | < 0.001 | < 0.05 |
| Starch in leaf | W7 (2) | < 0.01 | < 0.001 | 0.098 |
|  | W16 (11) | < 0.01 | < 0.05 | 0.293 |
| Glucose in (green) fruit | W7 (2) | n/a | n/a | n/a |
|  | W16 (11) | 0.054 | < 0.001 | 0.128 |
| Fructose in (green) fruit | W7 (2) | n/a | n/a | n/a |
|  | W16 (11) | < 0.05 | < 0.001 | < 0.01 |
| Sucrose in (green) fruit | W7 (2) | n/a | n/a | n/a |
|  | W16 (11) | < 0.001 | < 0.001 | < 0.05 |
| Fv/Fm | W7 (2) | 0.105 | < 0.001 | 0.187 |
|  | W16 (11) | 0.106 | < 0.05 | 0.780 |
| Φ_PSII_ at PFD300 | W7 (2) | < 0.001 | < 0.001 | < 0.001 |
|  | W16 (11) | < 0.01 | < 0.001 | < 0.05 |
| A_N_ at PDF300 | W7 (2) | < 0.001 | < 0.001 | < 0.01 |
|  | W16 (11) | < 0.01 | 0.131 | 0.185 |
| A_sat_ | W7 (2) | < 0.001 | < 0.001 | 0.230 |
|  | W16 (11) | < 0.001 | < 0.05 | 0.135 |

**
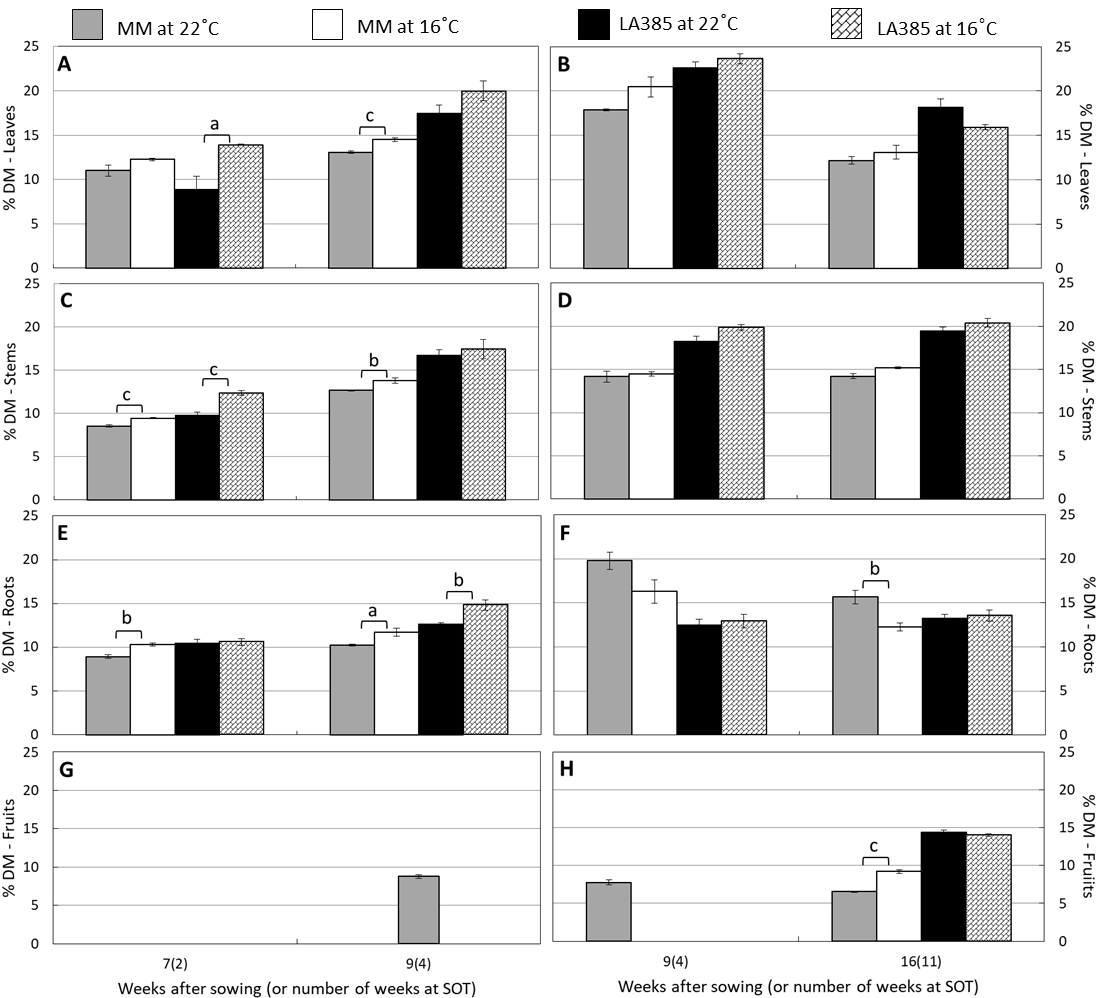
**

**Supplementary Figure 1.** Effect of SOT on dry matter content of different tomato organs of cv Moneymaker (MM) and wild species *S. arcanum* LA385. Panels on the left and right are from trial A and trial B, respectively. (n) is the number of weeks exposed to SOT. The error bars represent standard error of five individual plants (±SE), except week 9 of trial B with three plants. Significant difference is denoted as a, b, and c for *P* < 0.05, < 0.01, and < 0.001, respectively.

**
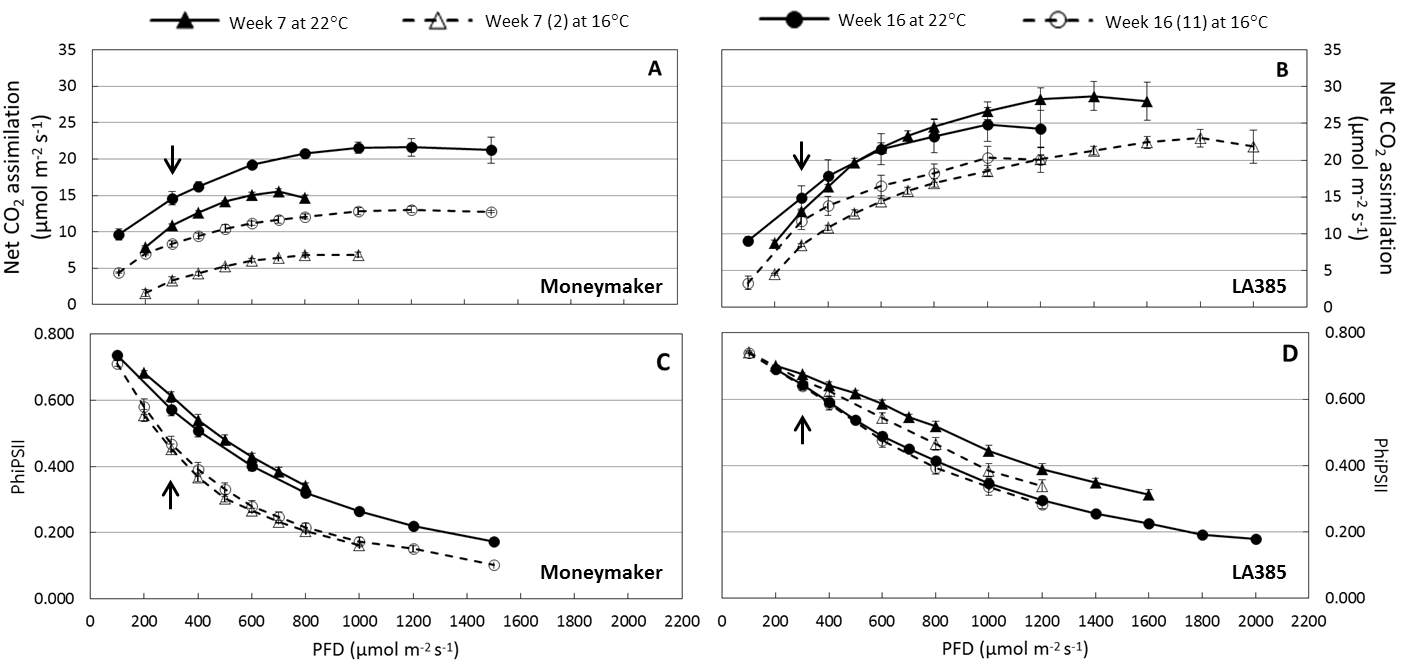
**

**Supplementary Figure 2.** Effect of SOT on photosynthetic parameters in week 7 (2) and week 16 (11) of cv Moneymaker and wild species *S. arcanum* LA385. Net CO_2_ assimilation rate (A_N_) and PhiPSII as a function of increasing light intensity starting from 0 till 2000 μmol m^-2^ s^-1^ at CO_2_ concentration of 400 μmol m^-2^ s^-1^ are demonstrated in panel A-B and panel C-D, respectively. We stopped measuring A_N_ with higher light intensity as soon as there was no more increase in A_N_ compared to the previous light intensity. *(n) is the number of weeks exposed to SOT. The arrows indicate the values at PFD of 300 μmol m^-2^ s^-1^. Data represent mean (±SE) of three to four plants.


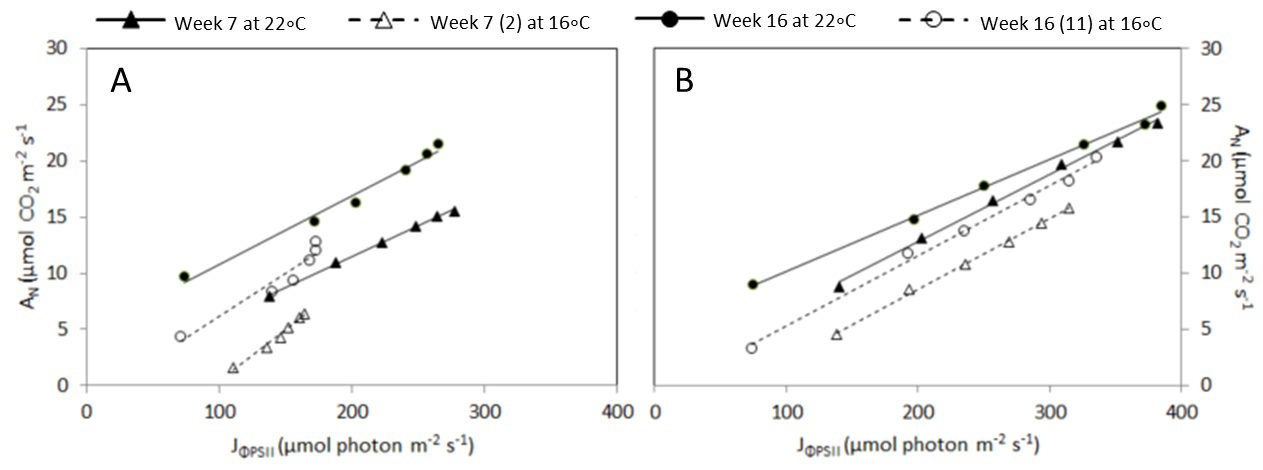


**Supplementary Figure 3.** Correlation between Φ_PSII_ and net CO_2_ assimilation rate. The correlation is shown as function of irradiance * Φ_PSII_ against CO_2_ fixation rate. The panel A and B show data of cv. Moneymaker and wild accession LA385, respectively. The data were derived from the calculation with irradiance from 200 – 1000 μmol photons m^-2^ s^-1^. The data represent mean of three to five independent measurements.
